# Iota-Carrageenan Is a Potent Inhibitor of Influenza A Virus Infection

**DOI:** 10.1371/journal.pone.0014320

**Published:** 2010-12-14

**Authors:** Andreas Leibbrandt, Christiane Meier, Marielle König-Schuster, Regina Weinmüllner, Donata Kalthoff, Bettina Pflugfelder, Philipp Graf, Britta Frank-Gehrke, Martin Beer, Tamas Fazekas, Hermann Unger, Eva Prieschl-Grassauer, Andreas Grassauer

**Affiliations:** 1 Marinomed Biotechnologie GmbH, Vienna, Austria; 2 Institute of Diagnostic Virology, Friedrich-Loeffler-Institut, Bundesforschungsinstitut für Tiergesundheit, Greifswald-Insel Riems, Germany; 3 St. Anna Children's Hospital, Vienna, Austria; 4 Laboratory of Tropical Veterinary Medicine, Veterinary University Vienna, Vienna, Austria; Hallym University, Republic of Korea

## Abstract

The 2009 flu pandemic and the appearance of oseltamivir-resistant H1N1 influenza strains highlight the need for treatment alternatives. One such option is the creation of a protective physical barrier in the nasal cavity. In vitro tests demonstrated that iota-carrageenan is a potent inhibitor of influenza A virus infection, most importantly also of pandemic H1N1/2009 in vitro. Consequently, we tested a commercially available nasal spray containing iota-carrageenan in an influenza A mouse infection model. Treatment of mice infected with a lethal dose of influenza A PR8/34 H1N1 virus with iota-carrageenan starting up to 48 hours post infection resulted in a strong protection of mice similar to mice treated with oseltamivir. Since alternative treatment options for influenza are rare, we conclude that the nasal spray containing iota-carrageenan is an alternative to neuraminidase inhibitors and should be tested for prevention and treatment of influenza A in clinical trials in humans.

## Introduction

While the current H1N1 influenza (flu) pandemic was ongoing in 2010, efforts were made to develop new antiviral agents for influenza treatment that possess an improved spectrum of activity or better pharmacologic profiles, compared to current treatments. The medical need for the development of new antiviral agents for the treatment of influenza virus-infected patients is mainly based on increasing resistance against currently approved drugs and on their limited antiviral efficacy in severe cases of influenza [1]. Available anti-influenza drugs target two different steps of the viral life cycle, the uncoating and the release of virus particles from infected cells. Uncoating of influenza A viruses is induced by the viral M2 ion channel protein and can be blocked by the adamantane-based compounds amantadine and rimantadine [2], [3]. Although clinically effective, these drugs caused considerable gastrointestinal and neurological side-effects in patients [4]. Moreover, emerging resistant influenza A viruses during seasonal influenza epidemics have been observed [5]. Today, the resistance level to amantadine has reached nearly 100% for H3N2-type influenza A virus strains, but resistant mutants are also frequently found among seasonal H1N1 isolates [1], [6]. Therefore, adamantanes are not considered anymore for routine use, but might be an option when all other measures fail [7]. The more recently approved antiviral agents to treat influenza infections are the neuraminidase inhibitors zanamivir and oseltamivir, both developed by rational drug design [8]. Influenza virus neuraminidase (NA) is anchored in the viral membrane and cleaves sialic acid-containing receptors on the surface of infected cells and on progeny virions. This enzymatic activity facilitates the movement of virus particles through the upper respiratory tract as well as the release (budding) of newly synthesized virions from infected cells [9]. Although highly efficacious in vitro [10] and in animal models [11], [12], in clinical trials neuraminidase inhibitors showed lower than expected efficacy against influenza symptoms in otherwise healthy adults [13]. However, in children with laboratory confirmed influenza, neuraminidase inhibitors were effective in reducing illness duration if given within 48 hours post exposure, but their efficacy in reducing severe complications in ‘at risk’ children, e.g. with asthma, awaits further investigation [14], [15]. Nonetheless, neuraminidase inhibitors have been used successfully as antiviral chemoprophylaxis for preventing and reducing the symptoms of seasonal influenza [16], [17]. Accordingly, in many countries neuraminidase inhibitors are stockpiled as means to prevent a worldwide pandemic [18], [19]. However, alternative treatment options are urgently needed as the current choice of drugs is limited and resistance is a constant threat [20].

One alternative approach to prevention and treatment of influenza is the creation of a protective physical barrier in the nasal cavity with carrageenans, high molecular weight sulphated polysaccharides derived from red seaweed (Rhodophyceae). Three main forms of carrageenans have been identified: kappa (κ), iota (ι), and lambda (λ). They differ from each other in sulphation degree, solubility and gelling properties [21]. Carrageenan is in widespread commercial use as an additive contributing to the texture and stability of various processed foods and cosmetic products, including some brands of sexual lubricant. Since high-quality carrageenan preparations (reviewed in [22]) appear to have a good safety profile for long-term use [23] and can inhibit HIV infections in model systems [24], clinical studies were conducted to validate the usefulness of carrageenan (Carraguard) as a vaginal microbicide for the prevention of HIV-1 transmission [24], [25]. Reasons for the failure of these studies are manifold and approaches to improve the efficacy of such topical formulations are in the focus of current research [26]. The antiviral potential of carrageenan and other sulphated polysaccharides in vitro against infections by several enveloped viruses such as herpes simplex virus (HSV-1 and HSV-2), human cytomegalovirus (HCMV), vesicular stomatitis virus (VSV), Sindbis virus, and human immunodeficiency virus has been described more than 20 years ago [27], [28], and has been reviewed recently [29]–[31]. Newer studies have confirmed the efficacy of carrageenans from different marine algal species in animal models of HSV and CMV infections in vivo [32]–[34]. The inhibitory mechanism of carrageenans on virus replication seems to comprise early events of the infection cycle, i.e. attachment and entry of virus particles [35], [36], and is dependent on the type of polysaccharide [29] as well as the serotype of the virus and the host cells [37].

Iota-carrageenan has been reported to inhibit the infection of certain non-enveloped human viral pathogens, e.g. human hepatitis A (HAV) and papilloma viruses (HPV) in vitro [37], [38]. Moreover, iota-carrageenan interfered specifically with the adsorption of HPV16 capsids to human sperm cells [39]. These findings encouraged the authors to propose clinical trials in order to determine whether carrageenan-based products are effective as topical microbicides against genital HPVs [38]. We recently could show that iota-carrageenan is a potent anti-rhinoviral substance in vitro and thus an ideal candidate for the treatment of infections that predominantly occur in the nasal cavity and upper respiratory tract [40]. Therefore, we were interested whether carrageenans and in particular iota-carrageenan have any antiviral activity against human influenza A viruses.

## Results

### Iota-carrageenan inhibits influenza virus-induced plaque formation on MDCK cells

We determined the sensitivity of the influenza virus strain H1N1 (A/PR8/34) and the formerly pandemic H3N2 (A/Aichi/2/68) to carrageenans of subtypes iota and kappa by plaque reduction assays in MDCK cells [41,41]. The results are summarized in Table 1. The two carrageenan types inhibited plaque formation of both viruses tested although to a varying degree. Iota-carrageenan was the most active substance in all experiments followed by kappa-carrageenan. The purity of iota- as well as kappa-carrageenan preparations analyzed by nuclear magnetic resonance spectroscopy (NMR) was greater than 95% and the molecular weight of both polymers well over 100′000 (data not shown). Nevertheless, the inhibitory potential of iota-carrageenan with IC_50_ values of around 0.2 µg/ml in H1N1 and 0.04 µg/ml in H3N2 infections was up to 10 times higher compared to kappa-carrageenan (Table 1). Generally, plaque formation by H3N2 viruses was inhibited at lower carrageenan concentrations when compared to H1N1. CMC, the control polymer, did not show any inhibitory effect up to the highest concentrations tested (400 µg/ml). No cytotoxicity of any of the polymers at the highest dosages was observed.

**Table 1:** Inhibitory effects of polysaccharide compounds.

Virus	IC_50_ * iota	IC_50_ kappa	IC_50_ CMC
A/Aichi/2/68 H3N2	0.04	0.30	>400
A/PR8/34 H1N1	0.20	2.70	>400

*Inhibitory concentration 50%: concentration in µg/ml required to inhibit influenza virus plaque formation on MDCK cells. Each value represents the mean of a quadruplicate assay.

### Iota-carrageenan promotes survival of influenza virus-infected MDCK cells and inhibits viral replication

**Figure 1:**
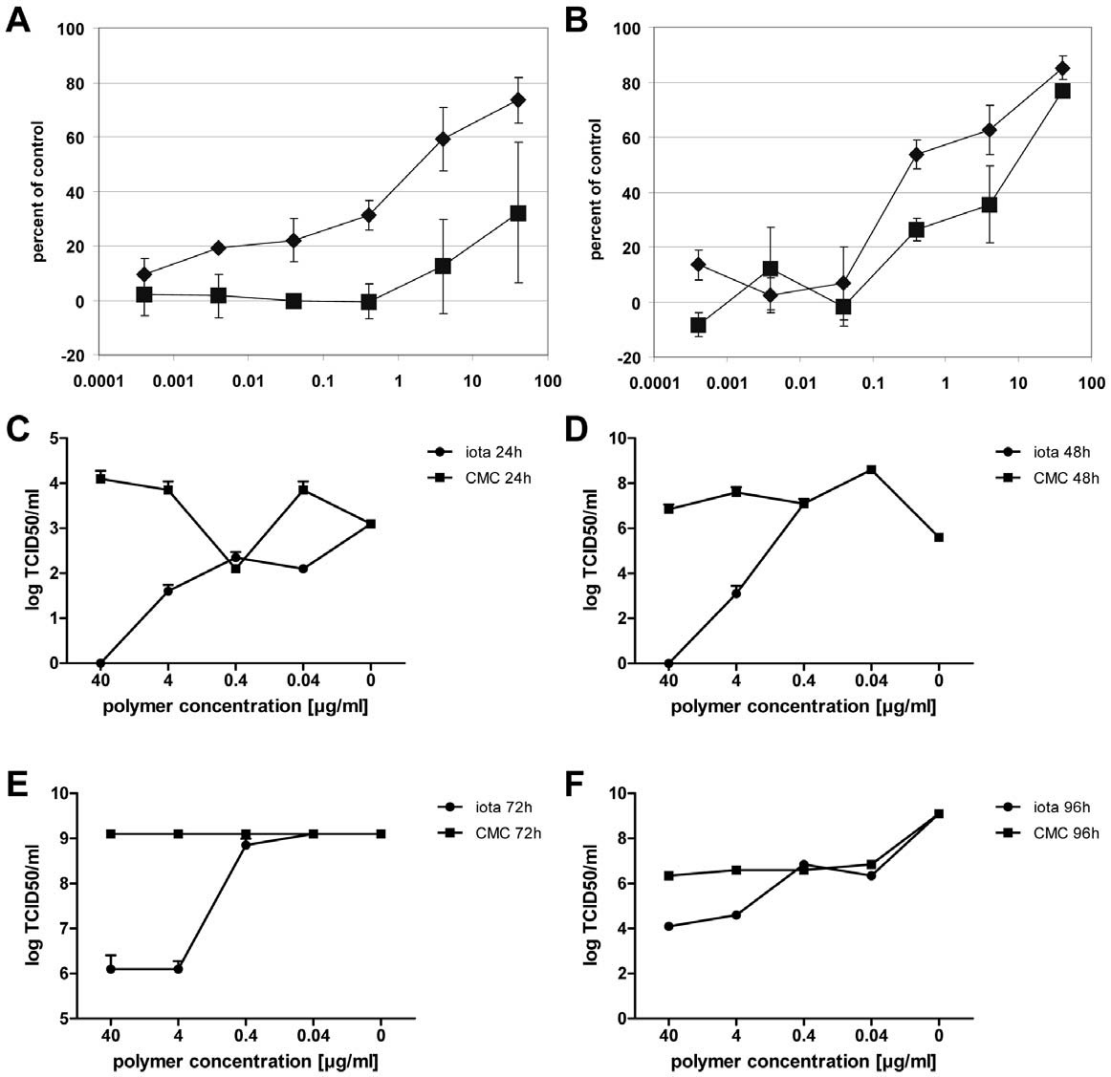
Iota-carrageenan promotes cell viability and reduces viral titer of influenza A-infected MDCK cells. MDCK cells grown in 96-well plates were infected with H1N1 A/PR/8/34 virus (A) and H3N2 A/Aichi/2/68 virus (B) (0.01 PFU/cell) in the presence of carrageenans (iota-carrageenan black diamonds, kappa-carrageenan black squares) at concentrations as indicated on the x-axis in µg/ml. Plates were incubated at 37°C until cells in the control (no polymer added) showed >90% damage. Cell proliferation was determined with a Resazurin-based in vitro toxicology assay. Samples were measured fluorometrically by monitoring the increase in fluorescence at a wavelength of 590 nm using an excitation wavelength of 544 nm. Values obtained from mock-infected cells were set to 100%, and the values of cells infected in the absence of polymer were set to 0% (y-axis). (C)-(F) MDCK cells were infected with H1N1 A/PR/8/34 as before and further kept in the presence of iota-carrageenan (circles) or the control polymer CMC (squares) at indicated concentrations and 24 (C), 48 (D), 72 (E), and 96 hours (F) post infection, respectively. Supernatants were harvested, pooled, and subsequently used to determine the TCID50/ml according to the method of Reed and Muench [59]. The points represent the mean of a quadruplicate experiment, the standard deviation is indicated.

MDCK cells were infected at low multiplicity (MOI = 0.01) with H1N1 A/PR/8/34 or H3N2 A/Aichi/2/68 virus in the presence of various concentrations of iota-carrageenan or CMC. After 48 hours, cell viability was determined with a standard cell proliferation test. A typical result obtained by this assay is shown in Figure 1. For instance, iota-carrageenan at a concentration of 4 µg/ml reduced the cytopathic effect of the A/PR/8/34 virus by more than 50% compared to control polymer CMC (Figure 1A). For cells infected with the A/Aichi/2/68 H3N2 virus, a concentration of 0.4 µg/ml of iota-carrageenan was sufficient to protect 50% of the cells from virus-induced cell death (Figure 1B).

In line with these findings, we have also determined the effect over time of different iota-carrageenan concentrations on viral replication of infected MDCK cells (Figure 1C–F). In marked contrast to the control polymer CMC, iota-carrageenan at concentrations of 40 and 4 µg/ml very efficiently reduced viral replication by 2–4 logs up to 96 hours post infection. Thus, iota-carrageenan efficiently promotes survival of influenza A-infected MDCK cells and does so by directly reducing the amount of virus released from infected cells.

### Iota-carrageenan promotes survival of influenza A/PR/8/34 virus-infected primary human nasal epithelial cells (HNep)

**Figure 2:**
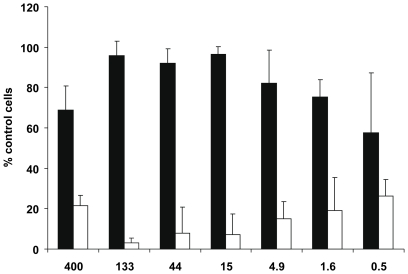
Effect of iota-carrageenan on influenza A-infected primary human nasal epithelial cells. Primary human nasal epithelial cells (HNep) cells grown in 96-well plates were infected with A/PR/8/34 virus (5 PFU/cell) in the presence of iota-carrageenan at concentrations indicated on the x-axis in µg/ml. 30 minutes after infection, the inoculum was removed and medium containing iota-carrageenan (black bars) or CMC (white bars) in indicated concentrations added. Cell proliferation was determined with a Resazurin-based in vitro toxicology assay. Samples were measured fluorometrically by monitoring the increase in fluorescence at a wavelength of 590 nm using an excitation wavelength of 544 nm. Values obtained from mock-infected cells were set to 100%, and the values of cells infected in the absence of polymer were set to 0% (y-axis). The bars represent the mean of a quadruplicate experiment, the standard deviation is indicated.

HNep cells were infected with influenza virus (5 PFU/cell) in the presence of iota-carrageenan or CMC as a control polymer and subsequently maintained in medium containing different amounts of polymers (0.5 µg/ml to 400 µg/ml). After 48 hours, cell proliferation was determined with a Resazurin-based in vitro toxicology assay. As shown in Figure 2, iota-carrageenan-treated cells showed significantly better protection from virus-induced cell death than cells treated with CMC at all concentrations tested. This result shows that iota-carrageenan promotes survival of influenza A virus-infected HNep cells.

### Iota-carrageenan is active against pandemic H1N1/2009 influenza virus in vitro

**Figure 3:**
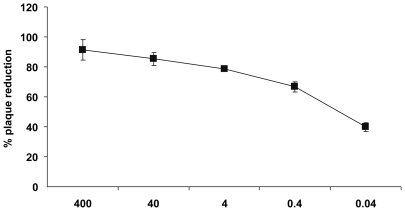
Effect of iota-carrageenan on pandemic H1N1/2009 virus. Confluent monolayers of MDCK cells in 6-well plates were washed free of protein-containing growth medium before use. An equal volume of virus suspension mixed with iota-carrageenan, containing 50 to 150 plaque-forming units, was added 5 to 10 min later, and plates were incubated at room temperature for 60 min with frequent shaking. The inoculum was removed and covered with an overlay medium consisting of 0.6% agarose (3 ml) in Eagle minimal essential medium and trypsin (2 µg/ml). Plates were incubated at 37°C in a humidified atmosphere with 5% CO_2_. After 36 to 48 h, plaques were stained with crystal violet and counted. The percentage of plaque inhibition relative to infected control plates (y-axis) was determined for each drug concentration (x-axis). The standard deviation of three independent experiments is indicated.

Since the A/PR/8/34 and A/Aichi/2/68 viruses were isolated several decades ago, we were interested whether iota-carrageenan bears antiviral activity also against the novel pandemic H1N1/2009 strain [18]. Similar to experiments with seasonal influenza virus strains, iota-carrageenan was found to strongly inhibit plaque formation of the pandemic H1N1/2009 strain in MDCK cells with an IC_50_ concentration of about 0.04 µg/ml (Figure 3). The IC_50_ values indicate that iota-carrageenan had the same antiviral potency against the pandemic H1N1/2009 strain as compared to the A/Aichi/2/68 H3N2 virus while inhibition of the A/PR8/34 H1N1 virus required five times higher concentrations of iota-carrageenan, at least in MDCK cells.

### Influenza A virus binding to iota-carrageenan beads is inhibited by dissolved iota-carrageenan but not by a control polymer

**Figure 4:**
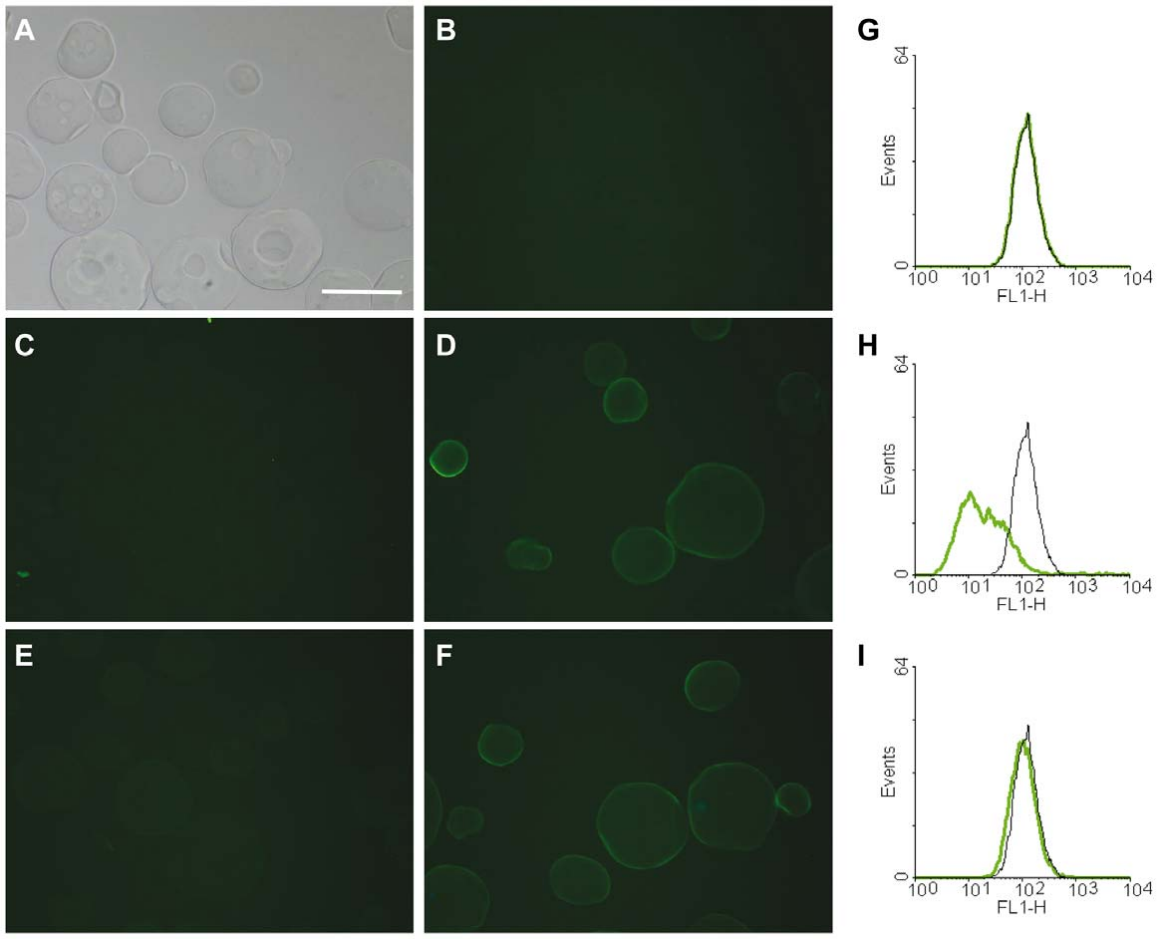
Binding of H1N1 influenza virus to iota-carrageenan. (A)-(F). Alexa Fluor 488-conjugated H1N1 influenza virus (H1N1-A488) was incubated with iota-carrageenan-coated agarose beads (iota-beads) or control beads for 30 min at room temperature and visualized microscopically. (A) Bright field picture of iota-beads, showing no green auto-fluorescence (B). (C) Control agarose beads incubated with H1N1-A488 do not facilitate unspecific virus binding. (D) Iota-beads incubated with H1N1-A488 demonstrates binding of virus to iota-carrageenan as evidenced by bright green staining of iota-beads. (E) Binding of H1N1-A488 to iota-beads is inhibited in the presence of iota-carrageenan (400 µg/ml), but is not abolished in the presence of CMC (400 µg/ml) (F). Scale bar  = 100 µm. (G) FACS analysis of MDCK cells incubated with H1N1-A488 in the presence of iota-carrageenan (400 µg/ml) (H) or control polymer CMC (400 µg/ml) (I) showing that binding of H1N1-A488 to cognate receptors is inhibited by iota-carrageenan but not CMC.

Several published reports indicate that the principal mechanism by which carrageenans block virus infectivity is by direct binding to the viral surface [35], [36], [38], [40]. In order to investigate whether a similar mechanism holds true for influenza viruses, we incubated iota-carrageenan-coated agarose beads with influenza A/PR/8/34 viral particles that were previously labelled with the fluorescent dye Alexa Fluor 488 (H1N1-A488). We found that the fluorescent virus directly binds to iota-carrageenan beads but not to agarose carrier material (Figure 4C, D). Importantly, binding of virus to iota-carrageenan was specific, as it was abolished in the presence of excess iota-carrageenan (Figure 4E), but not CMC (Figure 4F). Likewise, we independently confirmed this observation by using the same fluorescently-labelled H1N1 viral particles in FACS experiments with MDCK cells in the presence of iota-carrageenan or control polymer CMC. As shown in Figures 4G–I, only iota-carrageenan specifically competed with virus binding to MDCK cells but not CMC. These findings demonstrate that the antiviral mechanism of iota-carrageenan is conferred through direct binding of polymer to viral particles.

### Iota-carrageenan inhibits attachment of A/PR/8/34 influenza virus to cells

**Figure 5:**
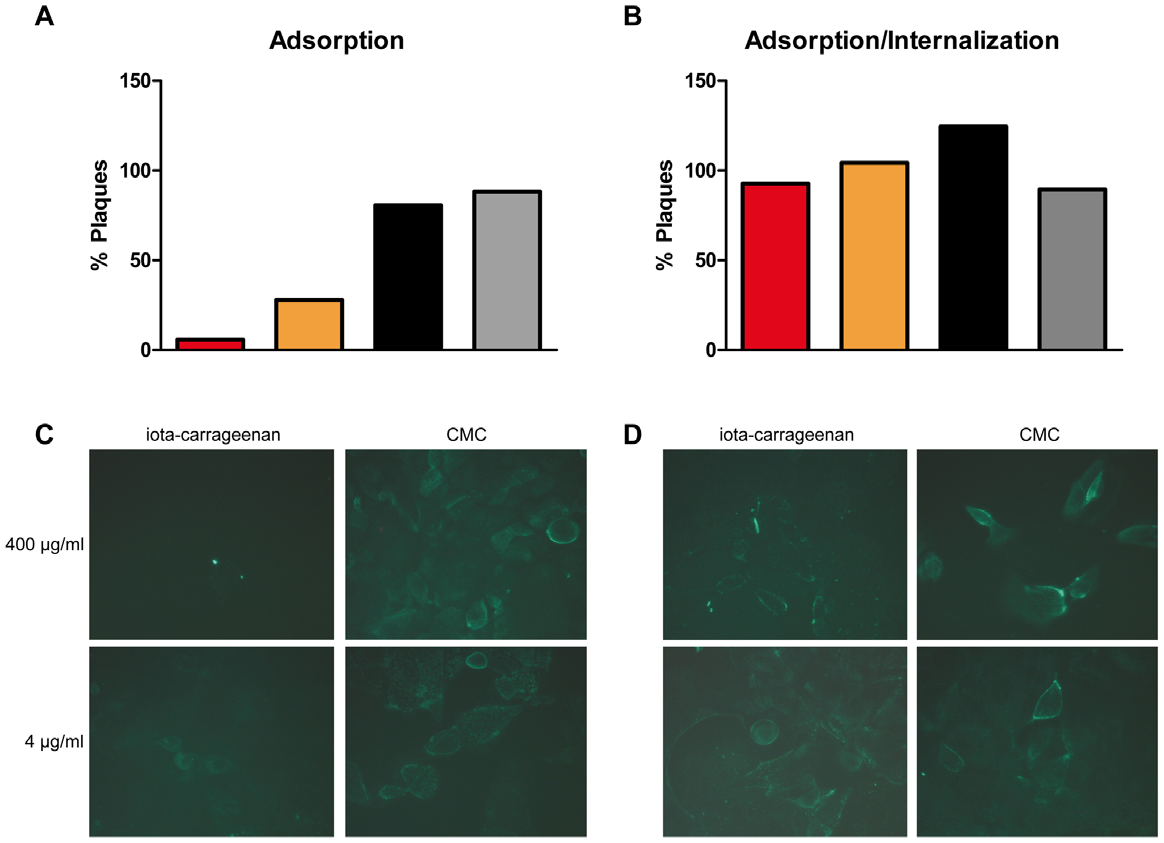
Effect of iota-carrageenan on H1N1 virus adsorption and internalization. (A) Adsorption. H1N1 virus was added to MDCK cells in the presence of different concentrations of iota-carrageenan or control polymer carboxymethylcellulose (CMC). After viral adsorption for 1 h at 4°C, cells were washed and the number of cell-bound infectious viral particles determined by plaque assay; red bar 400 µg/ml, orange bar 4 µg/ml iota-carrageenan, black bar 400 µg/ml, grey bar 4 µg/ml CMC. (B) Adsorption/Internalization. H1N1 virus was added to MDCK cells and adsorbed for 1 h at 4°C. Cells were washed and allowed to internalize virus in the presence or absence of different concentrations of iota-carrageenan or CMC for 1 h at 37°C. Subsequently, internalized infectious viral particles were determined by plaque assay. (C) Immunofluorescent visualisation of virus adsorption in the presence of iota-carrageenan or CMC. 1 h post adsorption at 4°C, cells were stained after 1 h at 37°C with a mouse anti-NP antibody. (D) Adsorption/Internalization. H1N1 was added to MDCK cells and adsorbed for 1 h at 4°C. Cells were washed and allowed to internalize virus in the presence of iota-carrageenan or CMC for 1 h at 37°C. Compare the bright green stainings in Figure 5D indicative of productive infection to 5C, where no green fluorescence is detected at high iota-carrageenan concentration.

To explore further the antiviral mode of action of iota-carrageenan, we performed time of addition studies in vitro. Therefore, iota-carrageenan was added to MDCK cells either before, after, or simultaneously with virus inoculum. The state of infection was analysed by plaque reduction assays (Figure 5, A and B) or alternatively, microscopically by staining the viral nucleoprotein (NP) with a monoclonal antibody (Figure 5, C and D). If iota-carrageenan was added to cells prior to infection, no positive effect on plaque reduction could be observed. Importantly, pre-incubation of cells with iota-carrageenan up to 48 hours was not toxic or altered proliferation of the cells in any way (data not shown). However, virus attachment to cells and hence, infection was dose-dependently blocked if iota-carrageenan was mixed with virus particles before addition to cells as evidenced in a reduction of formed plaques formed in MDCK cells and compared to control polymer (Figure 5A). Similar results were obtained with Vero cells (data not shown). In sharp contrast, if virus was allowed to adsorb to cells before addition of iota-carrageenan no protective effect on plaque formation similar to the control polymer CMC was observed (Figure 5B). When internalization of virus was assessed by immunofluorescence staining using an anti-NP antibody, infection of cells was only efficiently prohibited if virus was adsorbed in the presence of iota-carrageenan but not control polymer (Figure 5C) or if iota-carrageenan or control polymers were added post-adsorption (Figure 5D). In summary, iota-carrageenan inhibits influenza virus infection by directly interacting with virus particles thereby preventing adsorption to cellular receptors and subsequent internalization.

### Intranasal iota-carrageenan application significantly supports survival of mice infected with H1N1 A/PR/8/34 influenza virus

**Figure 6:**
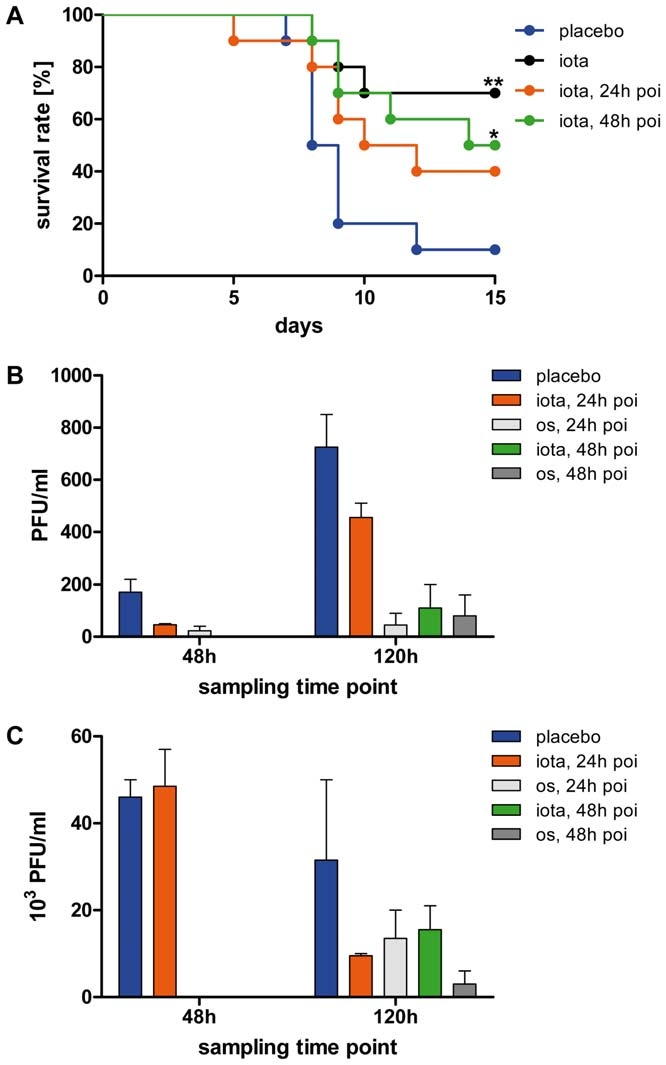
Therapeutic efficacy of iota-carrageenan against H1N1 influenza virus in a lethal mouse infection model. (A) Ten mice per group were intranasally infected with 8.7×10^2^ PFU H1N1 A/PR/8/34 viral particles at day 0. Intranasal therapy twice daily with 60 µg iota-carrageenan in 0.5% NaCl or placebo (blue) started on the same day as infection (black), 24 h post infection (poi) (orange), or 48 h poi (green), and was performed twice daily for the entire experiment. P values were calculated by a Log-rank (Mantel-Cox) test. Asterisk, p<0.05, double asterisk p<0.01. (B)-(C). Determination of viral titers from nose (B) and lung (C) specimens. Five mice per group were intranasally infected at day 0 as before. The group receiving placebo (blue) was compared to groups receiving intranasal therapy with iota-carrageenan or oral therapy with oseltamivir (10 mg/kg/day in 5% sucrose) starting 24 (orange or light grey) and 48 hours (green or dark grey) post infection until groups of mice were sacrificed at day 2 and 5 days, respectively. Subsequently, nose and lung specimens of animals from each experimental group and time point were pooled and viral titers determined by plaque assays on MDCK cells at two different dilutions. Bars represent the mean±SEM.

The pathogenicity of influenza viruses in mice varies and is dependent on the strain and its adaptation to its host. Depending on virus dose and strain, influenza virus can induce lethal infections in certain mouse strains usually within two weeks [42]. To investigate whether iota-carrageenan is efficacious in such a model, we challenged C57Bl/6 mice intranasally with a lethal dose of influenza H1N1 A/PR/8/34 virus and tested different treatment regimens in comparison to a vehicle control (placebo). Figure 6 illustrates one typical result of several independent experiments. If treatment with 60 µg iota-carrageenan per animal was started on day 0 immediately after infection and repeated twice daily during the course of the experiment (typically 15 days), we noticed significantly increased survival rates in the iota-carrageenan-treated group as compared to the placebo-treated control group. In the particular experiment shown in Figure 6, by day 15 after infection, 90% of the individuals in the placebo group had succumbed to the infection whereas 70% of the iota-carrageenan group had survived. In line with this result, survival rates were also significantly increased if treatment was started one day before infection (data not shown). Surprisingly, however, treatment could be started also one or two days after infection and still significantly increased survival rates. However, in contrast to other experiments performed during the course of this study, the difference between the 24 hours post-infection treatment schedule and the control group did not quite reach significance.

Intrigued by this finding, we conducted a separate experiment in which we determined the effect of intranasal iota-carrageenan treatment on viral titer of infected mice. We infected 5 mice per group as before and either started intranasal therapy with iota-carrageenan or oral therapy with oseltamivir 24 and 48 hours post infection as before, respectively. Subsequently, groups of mice were sacrificed 48 or 120 hours post infection and after semi-daily therapy and viral titers were determined from pooled specimens derived from the nasal cavity and lung by plaque assays. As shown in Figure 6B, intranasal treatment of mice with iota-carrageenan results in an immediate reduction of viral particles in the nasal cavity 2 days and even more pronounced 5 days post infection, in the same order of magnitude as the neuraminidase inhibitor oseltamivir. Conversely, while we could not determine a titer reduction in the lung 48 hours post infection (i.e. 1 day treatment) in the iota-carrageenan-treated group, we could clearly show a strong reduction of viral particles in the lungs of iota-carrageenan-treated mice 5 days post infection as compared to the control group (Figure 6B). Importantly, iota-carrageenan treatment seemed to be as efficient as an oseltamivir therapy and as before, we could see a benefit with respect of viral particle reduction in the nose and lung even if therapy was started as late as 2 days post infection. Intranasal therapy of infected mice with iota-carrageenan results in a survival benefit for mice and seems to be a direct consequence of a reduction in viral particles present in the nose and consequently in the lung at later time points of the infection, respectively.

### Oseltamivir and iota-carrageenan synergistically promote survival of mice in a therapeutic setting starting as late as 48 hours post infection

**Figure 7:**
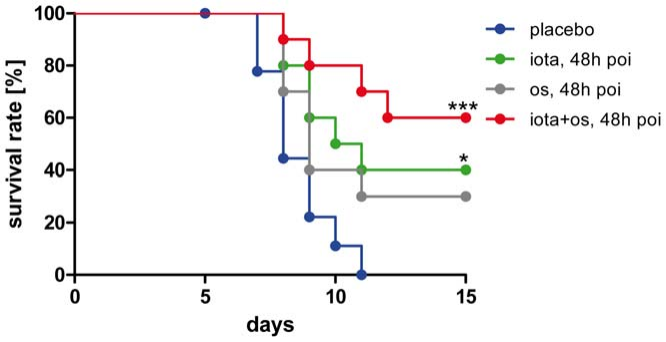
Efficacy of iota-carrageenan in mice in comparison to oseltamivir. Ten mice per group were intranasally infected with 8.7×10^3^ PFU H1N1/PR/8/34 viral particles at day 0 and therapy started 48 h poi (blue indicates the placebo treatment). In addition to the group with intranasal treatment twice daily with 60 µg iota-carrageenan (green), a group of mice also received an oral dose of oseltamivir (10 mg/kg/day in 5% sucrose) (grey) twice daily for 5 days, and accordingly in combination with iota-carrageenan (red). P values were calculated by a Log-rank (Mantel-Cox) test. Survival was monitored daily for 15 days. Asterisk, p<0.05; triple asterisk p<0.001.

To further explore the therapeutic potential of iota-carrageenan, we tested a combination with oseltamivir in the lethal infection model of C57Bl/6 mice but with a ten times higher virus dose as compared to the experiment shown in Figure 6. The treatment consisted of an intranasal application of iota-carrageenan (60 µg/animal/day in 0.5% NaCl) and an oral administration of oseltamivir (10 mg/kg/day in 5% sucrose). Treatment was started 48 hours post infection and repeated twice daily until the end of the experiment. Groups receiving oral doses of oseltamivir were treated for five days with this drug according to protocol, and thereafter received only iota-carrageenan and placebo intranasally as before, respectively. While iota-carrageenan alone in the particular experiment shown in Figure 7 supported the survival of infected animals at significantly increased rates, treatment with oseltamivir alone did not quite result in significantly higher numbers of survivors as compared to the placebo group. However, in other but similar experiments, oseltamivir monotherapy for five days at the same dose was efficacious, significantly and comparable to iota-carrageenan, if given 24 or 48 hours post infection (data not shown). Otherwise, all experiments with combination treatments showed the same effects, namely, significantly increased survival rates as compared to a monotherapy with either iota-carrageenan or oseltamivir.

## Discussion

In this report we demonstrate that iota-carrageenan, a biopolymer derived from red seaweed, is a potent inhibitor of influenza virus infectivity in vitro and in vivo. The report describes cell culture studies, demonstrates the antiviral activity of iota-carrageenan in mouse influenza infection models and proposes a mode of action.

The antiviral activity of iota-carrageenan against several virus types other than influenza has been studied more than 20 years ago. Antiviral activity was found against herpes simplex virus type (HSV) 1 and 2 at an IC_50_ of 2 and 10 µg/ml, respectively [28]. In the same report, iota-carrageenan was found ineffective against measles virus, adenovirus type 5, poliovirus and vesicular stomatitis virus. Our results indicate that iota-carrageenan is active against influenza A viruses at ten times lower concentrations when compared with HSV-1 in a standard plaque reduction assay (Table 1). This is comparable to our in vitro data of human rhinoviruses [40], but does not reach the low effectivity dosage range that has been described for papillomaviruses [38]. Both iota- and kappa-carrageenan protected MDCK cells from virus-induced cell death at an MOI of 0.01 (Figure 1A–B) in a dose-dependent manner. Moreover, maintenance of MDCK cells in the presence of iota-carrageenan up to 96 hours post infection with H1N1 also resulted in a dramatic reduction of viral titers by 2-4 logs, indicative of a protective effect of iota-carrageenan with regard to the spread and release of viral particles from previously infected MDCK cells (Figure 1C–F). However, an increased amount of input virus gradually reduces the protective effect. Therefore, we conclude that the antiviral effect of carrageenan is dependent on the relative amount of input virus in both cases. The data support the hypothesis that iota-carrageenan possesses antiviral activity due to direct interaction with the viruses.

To rule out tissue culture artefacts, we tested the antiviral activity of iota-carrageenan against influenza in primary HNep cells. The virus-induced CPE indirectly assessed by measuring cell proliferation showed that iota-carrageenan promoted cell survival at a concentration as low as 0.5 µg/ml (Figure 2). When compared to MDCK cells (compare Figure 1 with Figure 2), we found that iota-carrageenan showed a stronger antiviral effect on HNep cells. Since HNep cells are sensitive to trypsin, the assay was carried out at an MOI of 5 in the absence of trypsin. The CPE of HNep cells is therefore caused by a single replication cycle. Consequently, iota-carrageenan strongly inhibits the infection of HNep cells and the subsequent first round of infection, but would be less effective on cells already infected.

Importantly, iota-carrageenan had a similar antiviral effect on H1N1 and H3N2 virus infection of MDCK cells and Vero cells, respectively (data not shown). Since Vero cells have been previously described to be deficient in INF gene expression [43], the antiviral effect of iota-carrageenan is clearly not dependent on interferon. Collectively, the data obtained on MDCK, Vero and HNep cells suggest that iota-carrageenan interferes with viral replication at a very early stage of viral infection, i.e. viral adsorption and entry. Although iota-carrageenan binds to the cellular surface only weakly, its antiviral effect might be due to coating of cellular structures usually required for viral binding to its cognate receptors. In order to visualize this, we fluorescently labelled H1N1 virus and demonstrated that H1N1 directly binds to iota-carrageenan-coated agarose beads. Binding to iota-carrageenan was specific as it could be abolished in the presence of excess iota-carrageenan but not control polymer (Figure 4). When we studied the binding of fluorescently-labelled virus to MDCK cells by FACS, only iota-carrageenan specifically inhibited binding of labelled virus to cells (Figure 4). These results support the hypothesis that iota-carrageenan interferes with virus adsorption to the cells. When MDCK cells were treated with iota-carrageenan after adsorption of influenza virus to cells, we did not observe plaque reduction as well as reduction of the signal when stained with a NP-specific antibody, respectively. Therefore, iota-carrageenan does not prevent the virus from being internalized once it successfully binds to its receptor. In contrast, when iota-carrageenan was already present during viral adsorption, a strong reduction in plaque counts was observed and no signal could be detected in immunofluorescence stainings for influenza-specific NP protein (Figure 5). These findings lead us to the conclusion that the antiviral effect of iota-carrageenan differs in dependence of the virus. Recent data obtained with Dengue virus showed that carrageenan may interfere not only with adsorption of virus to cells but also block the fusion event leading to uncoating of the nucleocapsid [36]. In contrast, our data obtained with influenza virus demonstrate that iota-carrageenan exerts its antiviral effect by effectively inhibiting virus adsorption to host cells and hardly seems to interfere with later stages of the viral life cycle.

The recent outbreak of the pandemic (H1N1) 2009 virus continues to expand in humans particularly in people at risk, such as elderly or immuno-compromised individuals [44]. Thus, it was important to determine whether iota-carrageenan has a similar effect against the current pandemic (H1N1) 2009 virus strain. As shown in figure 3, iota-carrageenan is highly active against the current pandemic strain at similar concentrations as compared to A/Aichi/2/68 H3N2 virus while inhibition of the A/PR8/34 H1N1 virus required five times higher concentrations of iota-carrageenan (see Table 1). Given that pandemic H1N1/2009 virus might persist in the population for several decades, it is of great importance to have an effective treatment alternative with iota-carrageenan, which might become very useful in case resistencies of pandemic H1N1/2009 against the neuraminidase inhibitors oseltamivir or zanamivir develop.

Mice are a well accepted animal model for the development of antiviral compounds against influenza [42]. The susceptibility of mice to pandemic (H1N1) 2009 virus has been shown to be limited [45]. Consequently, we referred to established mouse models based on known influenza virus strains. Data obtained with A/Aichi/2/68 virus in a non-lethal animal model indicated that therapy with iota-carrageenan indeed had a positive effect on animal weight during infection (data not shown). Encouraged by these results we switched to a lethal mouse model based on the A/PR/8/34 virus. In this model, mice were infected intranasally with a lethal dose of virus without the utilization of narcosis. This procedure ensured that virus and the therapeutic solution were applied to the nose and not directly to the lung. As shown in Figure 6A, semi-daily intranasal treatment with a 1.2 mg/ml iota-carrageenan solution resulted in significant improvements of survival rates. While mice showed the best survival rate when treatment was started immediately after infection, we observed that iota-carrageenan treatment significantly promoted survival of infected animals even when treatment was started as late as 48 hours after infection. There was no statistical difference between mice whose treatment was started 24 hours or 48 hours after infection. Statistical power calculations of the experiments revealed that an unethically large population size would be necessary to determine a statistical difference between these two treatment groups.

Guided by our in vitro data which clearly suggest a reduction of viral particles released from infected cells in the presence of iota-carrageenan, we predicted that the same would hold true in the in vivo infection model thereby arguing that the significantly increased survival rate in the iota-carrageenan-treated group could be attributed to a reduction of viral particles present in the animals. To specifically address this issue, we performed another animal experiment in which we sacrificed animals at certain time points post infection (48 and 120 hours) and semi-daily treatment with intranasal iota-carrageenan or oral oseltamivir. Importantly, we started the therapy 24 or 48 hours post infection as to account for a realistic treatment regimen for future patients thereby assuming that patients would most likely start therapy shortly after realizing first signs of a flu infection (i.e. 24–48 hours post infection). As iota-carrageenan was applied intranasally, we were obviously interested to determine the viral titer within the nose and reasoned that a reduction in the upper respiratory tract by iota-carrageenan treatment should consequently translate into less virus spreading from the upper respiratory tract to the lungs (from which the animals ultimately die due to severe lung inflammation). As summarized in Figure 6B, we were able to convincingly demonstrate that application of iota-carrageenan to the nose seems to almost instantaneously translate into a reduction of viral particles in the nose, i.e. 48 hours post infection and one semi-daily therapy starting 24 hours after the initial infection. This effect got more pronounced 5 days after the initial infection, even when we started therapy 48 hours thereafter (i.e. semi-daily therapy for 3 days). As for viral spread to the lungs (Figure 6C), we were not able to see a titer reduction in the iota-carrageenan-treated group at the 48 hour sampling point, in sharp contrast to the orally treated oseltamivir group. However, at the second sampling point 5 days post infection, iota-carrageenan-treated animals had dramatically reduced lung titers as compared to the placebo group and in the same order as the oseltamivir group. Taken together, we propose that intranasal iota-carrageenan treatment within a short time frame very efficiently counteracts viral replication in the upper and spread to the lower respiratory tract thereby providing a rationale as to why intranasal iota-carrageenan treatment translates into a survival benefit as opposed to placebo-treated animals. Those experiments do not sufficiently address the question if the survival benefit of iota-carrageenan-treated animals is due solely to a reduction of viral particles spreading from the nose to the lung, or if other effects also contribute that have not been addressed so far. Iota-carrageenan has a molecular weight above 500 KDa, does not to cross mucosal membranes and did not show any inhibitory or stimulatory effects on a panel of immune cells (data not shown). We conclude that due to a direct interaction of virus with polymer, binding of virus to cells is hindered. Consequently, we speculate that the subsequent viral replication-induced innate response of the host is reduced and the survival of the animals is promoted. This is further substantiated by our own findings in an exploratory study in volunteers with early symptoms of the common cold [61], in which intranasal administration of iota-carrageenan reduced the symptoms of common cold (p = 0.046), viral load in nasal lavages (p = 0.009), and amongst other cytokines also IL-8. However, it is difficult to assess if that finding is a direct or indirect effect in patients and clearly awaits further experimental analysis in future clinical trials.

In order to further substantiate the above finding, we increased the viral dose 10-fold and compared the antiviral efficacy of iota-carrageenan to oseltamivir and a combination of both drugs, respectively (Figure 7). The results of the experiment suggest that iota-carrageenan promotes survival of influenza A-infected mice even when treatment is started after 48 hours and the viral dose increased 10-fold when compared to Figure 6A. There was no statistically significant difference between the treatments with iota-carrageenan and oseltamivir alone. However, when we combined iota-carrageenan and oseltamivir and started the treatment 48 hours post infection, 60% of the mice survived the lethal influenza dose. This result suggests that iota-carrageenan and oseltamivir show additive therapeutic effects when given in combination up to 48 hours post infection in mice.

The therapeutic use of neuraminidase inhibitors is broadly described in the literature. As reviewed by Burch et al. [46], the overall benefit of neuraminidase inhibitors in influenza virus-infected adults is primarily seen in a reduction of the average time period between the occurrence of the first disease symptoms of infection and the beginning of symptom alleviation in influenza virus-infected adults. For example, the administration of the antiviral drug zanamivir to infected patients of the non-risk adults group may reduce the median value for the time interval to detectable symptom alleviation by 0.57 days, while the administration of oseltamivir achieves a reduction by 0.55 days. These data suggest that there may be a need for improved therapeutic strategies based on compounds such as iota-carrageenan. Of concern, however, is that widely-used monotherapy with oseltamivir for the treatment of seasonal influenza has already selected a considerable proportion of resistant variants among circulating influenza A (H1N1) strains [46]–[48]. Rapid global dissemination of a H1N1 strain carrying a resistance-conferring neuraminidase (NA) gene with an H274Y amino acid substitution occurred during the 2007/2008 flu season [46], [49] similar to the previously observed emergence and fast spread of amantadine-resistant H3N2 strains [5], [6]. In contrast to expectations from earlier studies pointing to a reduced viability of H274Y-mutant strains [50], [51], recent clinical isolates showed an unimpaired replication potential in vitro and full virulence in vivo [52]–[55]. However, the seasonal H1N1 strain circulating since 2008 differs at several positions in the NA gene other than H274Y and is therefore considered as a natural variant of previous strains [56], [57]. As the influenza virus life cycle critically depends on a balance between available receptor sites (neuraminidase) and receptor binding (hemagglutinin), the new variant may have emerged by selection of a compensatory co-mutation in the hemagglutinin gene to acquire full virulence [9], [56].

Polysaccharides and in particular carrageenans were found to be potent antiviral agents against certain viruses. The antiviral effects of carrageenans were of limited practical importance so far, most likely because carrageenans are high-molecular weight components making it unlikely that they pass the different barriers of the body or even the cell membrane. However, these characteristics do not rule out local applications. A recent study with Carraguard, a carrageenan-based compound developed by the Population Council, did not show efficacy in prevention of vaginal transmission of HIV [26]. The authors conclude that low acceptance of gel use could have compromised the potential to detect a significant protective effect of Carraguard. In contrast to influenza viruses, HIV causes a persistent systemic infection that is usually not cleared by the immune system of the organism. Therefore, an incomplete protective effect at the entry site of the virus might lead to full blown HIV infection that is inaccessible to treatment with an antiviral polymer.

The results of our animal experiments allow the speculation that treatment with iota-carrageenan reduced the spreading of influenza virus in surface epithelia of infected animals and thereby provided sufficient benefit for animals to promote survival. In conclusion, our results suggest that iota-carrageenan is safe and effective in treating influenza infection in an animal model. Moreover, given that a iota-carrageenan-containing nasal spray is already marketed in Europe and has successfully been tested in an exploratory trial for treating common cold in humans [61], iota-carrageenan is also a promising antiviral candidate for the prophylaxis and treatment of influenza virus infections and should be tested for prevention and treatment of influenza A in clinical trials in humans.

## Materials and Methods

### Compounds

Kappa-carrageenan and iota-carrageenan were purchased from FMC Biopolymers (Philadelphia, PA). The dry polymer powders were dissolved in cell culture water (PAA, Austria) to a final concentration of 0.4%. This stock solution was sterile filtered through a 0.22 µm filter (Sarstedt, Germany) and stored at 4°C until use. The identity and purity (>95%) of carrageenan subtypes was confirmed by NMR analysis as described elsewhere [58]. It should be noted that commercially available batches of lambda-carrageenan contained up to 30% of iota- and/or kappa-carrageenan and others. Therefore, we excluded lambda-carrageenan preparations in this study. Oseltamivir was extracted from Tamiflu capsules (Roche, Switzerland).

### Cells and viruses

Madin-Darby canine kidney (MDCK) cells were obtained from the American Type Culture Collection (Manassas, VA) and grown in DMEM supplemented with 10% fetal calf serum (FCS; PAA, Austria). Human nasal epithelial cells (HNep) were obtained from PromoCell GesmbH (Heidelberg, Germany) and cultivated in airway epithelial cell growth media (PromoCell). Vero (embryonic African green monkey kidney) cells were purchased from the American Type Culture Collection (ATCC; Manassas, Va.) and grown in serum-free medium (Invitrogen, Darmstadt Germany). Viruses A/PR/8 (H1N1) and A/Aichi/2/68 (H3N2) were purchased from ATCC. Pandemic H1N1/2009 Virus A/Regensburg/D6/09/H1N1 was kindly provided by Stephan Becker, University of Marburg, Germany. All influenza viruses were propagated in MDCK cells.

### Anti-influenza activity

Anti-influenza virus activity was evaluated by plaque reduction assays. Confluent monolayers of MDCK cells in six-well tissue culture plates were inoculated with 70-120 PFU of virus per well. After 60 min, the inoculum was removed and the test medium was added. MDCK cells inoculated with influenza A viruses were incubated under 100% humidity and 5% CO2 in a 0.5% agarose medium containing 0.001% of 2 µg of trypsin per ml for 2 days at 35°C. The cells were stained with 0.005% crystalviolet solution and the plaque numbers were counted. The 50% inhibitory concentrations (IC_50_s) were determined as the concentrations required to reduce the number of plaques to 50% of the number in wells containing no compounds.

The 50% tissue culture infectious dose (TCID_50_) was determined in MDCK cells with 10-fold serially diluted viruses incubated at 37°C for 72 hours. Virus titers in 50% tissue culture infectious doses (TCID_50_)/ml were determined according to Reed and Muench [59].

### CPE inhibition assays

For determination of antiviral activity, a CPE inhibition assay was performed. MDCK cells were seeded in tissue culture plates 24 hours prior to the experiments. At 80% confluence cells were infected with inoculums at defined amounts of input virus (TCID_50_/cell). In order to test whether the polymers can inhibit viral infection the cells were infected with virus in the presence or absence of polymer. For determination of virus-induced reduction in cell viability the cell metabolism was measured with an alamarBlue assay (AbD Serotec, Duesseldorf, Germany). The relative fluorescence at 544 nm (emission wavelength 590 nm) was determined in an Omega reader (BMG Labtech, Offenburg, Germany).

Relative cell survival in the presence of inhibitors was calculated by setting mock-infected cells to 100% survival and cells infected without inhibitor to 0% survival.

### Alexa Fluor 488 labelling of virus

Labelling of virus was adapted from a protocol described elsewhere [60]. Briefly, A/PR8/34 virus was grown in MDCK cells and concentrated to 10^5^ HAU via sucrose gradient purification. 1×10^4^ HA units of virus in a given volume were incubated with 1:10 of 1.0 M sodium bicarbonate pH 9. Alexa 488 was dissolved in 200 µl CMS to a final concentration of 5 µg/µl and was added in a ratio of 0.005 µg/HAU. The virus-Alexa mixture is wrapped into tinfoil and placed on a rotation mixer for 1 hour. Afterwards, the sample is dialyzed against PBS at 4°C. HA titre was controlled via HA test and infectious particles were determined by plaque assay to be 4*10^8^ pfu/ml.

### Immunocytochemistry

Iota-carrageenan beads (#02893) were provided by BioScience Beads Division (West Warwick, RI). Beads were washed with PBS mounted on glass slides and then treated with an Alexa488-conjugated Influenza H1N1 PR8/34 virus. Alternatively, beads were pre-incubated with 400 µg/ml iota-carrageenan, washed and incubated with the conjugated virus. The beads were examined in a Nikon Diaphot Fluorescence Microscope with a 61001 filter (D/F/PI; C 121798). Images were taken by a CCD-camera and analysed by Nikon NIS elements. The virus attached to the beads showed bright green staining. As negative control beads were analysed without virus incubation.

MDCK cells were grown in chamber slides in DMEM medium with 10% FCS, washed in medium and incubated for the adsorption experiment with a mixture of either iota-carrageenan (400 or 4 µg/ml) and H1N1 (MOI = 1) or CMC (400 or 4 µg/ml) and H1N1 virus (MOI = 1) for 1 h at 4°C in medium. For the adsorption/internalization experiment, MDCK cells were incubated with virus for 1 h at 4°C prior to incubation with iota-carrageenan or CMC (1 h, 37°C). For both experiments cells were washed afterwards with medium and fixed with 1% formaldehyde. Cells were permeabilized with 0.3% TritonX-100, 10% FCS in PBS for 15 min at RT, incubated with anti-NP in PBS with 10% FCS, 0.1% Tween20 (dilution: 1∶200; mouse anti-influenza A (anti-NP), AbD Serotec, 0300-0234) for 1 h at RT, washed with PBS with 10% FCS, 0.1% Tween20 and then incubated with an anti-mouse Alexa 488-conjugated secondary antibody (Invitrogen, Germany) diluted 1∶200 in PBS with 10% FCS, 0.1% Tween20 for 1 h at RT. Cells were examined microscopically by the same equipment as described above. As negative controls cells without any treatment, cells with primary or secondary antibody only, and cells without virus-incubation but antibody treatment were used. All negative controls showed no staining (not shown).

### FACS analysis

For FACS analysis, 1×10^6^ MDCK cells per sample were used. Cells were detached with 0.5 mM EDTA, incubated with H1N1-A488 (MOI = 1) in the presence (or absence) of iota-carrageenan and CMC as control polymer (each 400 µg/ml), washed with PBS, fixed with 1% formaldehyde and examined directly afterwards with a BD-FACSCalibur and analysed with WinMDI2.9. Negative controls included cells only and cells with free Alexa488 (not shown).

### Ethics Statement

All animals were handled in strict accordance with the guidelines of the “European Convention for the Protection of Vertebrate Animals used for Experimental and other Scientific Purposes” and the Austrian law for animal experiments (BGBl. Nr. 501/1989 idF BGBl. I Nr.162/2005). All experimental procedures were discussed and approved by the Veterinary University of Vienna institutional ethics committee and performed under the Austrian Federal Ministry of Science and Research experimental animal license numbers BMWF-68.205/0130-II/10b/2008 and BMWF-68.205/0135-II/10b/2009.

### Pathogenicity and lethality in C57BL/6 mice

All mice were maintained under standard laboratory conditions in the animal facility of the Veterinary University of Vienna. To determine the 50% mouse lethal dose (MLD_50_) groups of 10 female 6-week-old C57BL/6 mice (Charles River Laboratories, Sulzfeld, Germany) were intranasally inoculated with 50 µl of 10-fold serial dilutions of A/PR8/34 (H1N1) virus in tissue culture medium without narcosis. The MLD_50_ values were calculated after a 14-days observation period by the Reed-Muench method [59]. The determined values for 1 MLD_50_ of the viruses were expressed as PFU. To determine the viral titer of lung and nose samples, tissue samples were snap-frozen upon dissection and subsequently homogenized in serum-free DMEM medium for plaque reduction assays as described above.
